# Lifetime Prevalence and Factors Associated with Head Injury among Older People in Low and Middle Income Countries: A 10/66 Study

**DOI:** 10.1371/journal.pone.0132229

**Published:** 2015-07-06

**Authors:** A. Khan, M. Prince, C. Brayne, A. M. Prina

**Affiliations:** 1 Institute of Public Health, University of Cambridge, Forvie Site, Robinson Way, Cambridge, CB2 0SR, United Kingdom; 2 King’s College London, Institute of Psychiatry, Psychology and Neuroscience, Health Service and Population Research Department, Centre for Global Mental Health, De Crespigny Park, London, SE5 8AF, United Kingdom; Yokohama City University, JAPAN

## Abstract

**Introduction:**

Traumatic brain injury (TBI) is a growing public health problem around the world, yet there is little information on the prevalence of head injury in low and middle income countries (LMICs). We utilised data collected by the 10/66 research group to investigate the lifetime prevalence of head injury in defined sites in low and middle income countries, its risk factors and its relationship with disability.

**Methods:**

We analysed data from one-phase cross-sectional surveys of all residents aged 65 years and older (n = 16430) distributed across twelve sites in eight low and middle income countries (China, Cuba, Dominican Republic, India, Venezuela, Mexico, Peru, and Puerto Rico). Self-reported cases of head injury with loss of consciousness were identified during the interview. A sensitivity analysis including data provided by informants of people with dementia was also used to estimate the impact of this information on the estimates. Prevalence ratios (PR) from Poisson regressions were used to identify associated risk factors.

**Results:**

The standardised lifetime prevalence of TBI ranged from 0.3% in China to 14.6% in rural Mexico and Venezuela. Being male (PR: 1.6, 95% CI: 1.29–1.82), younger (PR: 0.95, 95% CI: 0.92–0.99), with lower education (PR 0.91, 95% CI: 0.86–0.96), and having fewer assets (PR 0.92, 95% CI: 0.88–0.96), was associated with a higher prevalence of TBI when pooling estimates across sites.

**Discussion:**

Our analysis revealed that the prevalence of TBI in LMICs is similar to that of developed nations. Considering the growing impact of TBI on health resources in these countries, there is an urgent need for further research.

## Introduction

Traumatic brain injury (TBI) is a major global public health problem, with around 1.7 million cases recorded annually in the USA alone, corresponding to approximately 0.5% of the country's population[1]. TBI is generally defined as physical damage to the brain that is commonly characterised as mild, moderate, or severe, and is heterogeneous in its diagnosis, treatment, and prognosis[2]. Mild TBI, which is often used interchangeably with concussion is the most common head injury, accounting for approximately 70–90% of all TBI cases[3]. However, concussion itself is not well defined in either clinical or research contexts[4].

A recent meta-analysis reported a 12% estimate of a lifetime history of TBI with loss of consciousness among adults (aged 18 and over) living in the community [5]. All the included studies were however conducted in Australia, North America and Canada, highlighting the lack of estimates from low and middle-income countries (LMIC). In the USA, TBI occurs predominantly in people over 65 years of age, and children under 4 years. Falls are the leading cause of TBI in the USA and occur predominantly in younger and older age groups. A second peak in TBI emergency visits and hospitalisations is also found at age 19, which is driven by motor vehicle crashes [1]. Men are generally more likely to suffer TBI than women[1, 5]. Estimates of TBI are similar in other European countries, but could be higher in hospital settings, with one million cases of TBI recorded in hospitals across 23 European nations each year[6], [7]. It has been shown that the number of older people with a history of TBI is increasing[8].

For most low and middle income countries the incidence of TBI is thought to be similar as that of high-income nations, although this is based on a review of a very small number of studies[6]. Road traffic accidents are the leading cause of TBI in less developed regions, accounting for almost 60% of all cases, with falls making up only 20–30[6, 9, 10]. Due to increasing use of motor vehicles, continuing urbanisation, and population ageing the number of TBI cases in LMIC is expected to continue to rise[6]. Globally, the World Health Organisation predicts that TBI will become one of the leading causes of disability and death by the year 2020[6]. In addition, old age is a major risk factor for falls and therefore TBI, with the fatality rate of TBI increasing from 20% in childhood to 71% for over 75-year-olds[11]. In the elderly falls can result from gait impairment, a history of stroke, cognitive impairment, or poor vision[12]. TBI in older adults is a particular problem because age can negatively affect the outcome of the injury[13, 14]. In the cases where TBI is not lethal, it can result in physical and cognitive disability, leading to functional and social problems[15, 16]. People with TBI may also be at higher risk of harming themselves and of psychiatric illness[17, 18].

TBI and its related outcomes have increasing social and economic costs that have to be paid for by both public and private healthcare systems[19]. Although TBI is a rapidly growing public health problem in LMICs, few studies have been carried out in these settings[20]. Existing data has been derived chiefly from studies of samples from developed countries [21].

The key aim of this work it to investigate the prevalence of head injury with loss of consciousness among older adults in a number of LMICs belonging to the 10/66 population-based study, using a standardised methodology. We also aim to investigate some of the risk factors of head injury, and explore its relationship with disability.

## Methods

### Settings and sample

Details of the 10/66 methods have previously been published[22]. The 10/66 Dementia Research Group was established in the 1990s to address the gap in research in developing countries by encouraging high quality research into dementia, ageing, and non-communicable diseases in these countries. In brief, cross-sectional surveys of all people who were aged 65 or over were carried out in defined study catchment areas located in Cuba, Dominican Republic, Venezuela, Mexico, Peru, India, China, and Puerto Rico, with each country interviewing around 2000 subjects in order to allow estimation of a typical dementia prevalence of 4.5% (SE 0.9%) with 80% power.

Recruitment and assessment were carried out between 2003 and 2007. Boundaries for each catchment area were precisely defined. For urban catchment areas, populated by mainly middle-class professionals, areas with high-income earners were avoided. Rural catchment areas were defined as having low population density, and traditional lifestyle. All community-residents aged 65 and over within each catchment area were approached by means of door-knocking using a process of full household enumeration. The only exclusion criteria was being younger than 65.

### Interviews and measures

Each centre was allocated a project coordinator who supervised between 4–10 interviewers. All of the assessment tools were professionally translated into the relevant local languages (Ibero-American Spanish, Tamil, and Mandarin) and supplemented by the provision of video training materials. Field interviews were regularly checked and supervised.

Interviews were completed in participants’ residences and consisted of a comprehensive assessment that lasted between 2–3 hours, including the measurement of lifestyle and socio-economic factors. Age was confirmed by the interviewer from official documentation and informant reports. Discrepancies were resolved through further questions and clarification and, ultimately, by consensus within the research team. Level of education (none/did not complete primary/completed primary/secondary/tertiary), number of household assets (car, television, refrigerator, telephone, plumbed toilet, water, and electricity mains) and alcohol problems in mid-life (self-reported) were also recorded.

Disability was measured using the WHO-DAS II, developed by the World Health Organisation as a culture-fair assessment tool for use in cross-cultural epidemiological and health services research[23].

### Head injury ascertainment

Head injury exposure, here defined as "head injury with loss of consciousness" was ascertained by asking the participant the following question: “have you ever had a serious head injury in which you were knocked out”. Age of injury was also assessed. For participants who showed signs of dementia, based on a score of 2 or more on the CSI-D informant RELSCORE[24], an informant questionnaire used as part of the 10/66 dementia diagnostic assessment for evidence of cognitive and functional decline, were screened with the History and Aetiology Schedule-Dementia Diagnosis and Subtype (HAS-DDS). This is an extended informant interview that includes more detailed information on the onset and course of possible dementia. During the interview, the informant was asked whether “has your (xxx) ever had a serious head injury in which your (xxx) was knocked out”? This information was used in a sensitivity analysis to explore the impact that adding this data would have on the prevalence estimates. If an individual had more the one episode of head injury, only the latest episode was recorded. The number of multiple episodes of head injury throughout the life course was not recorded.

### Statistical Analysis

For each centre the key characteristics of the participants were described including age, sex, marital status, educational level, income, and number of assets.

Lifetime prevalence of head injury stratified by gender and with robust 95% confidence intervals adjusted for household clustering was reported. To improve comparison of data between study centres, direct standardisation for age, gender and education using the whole sample from all countries as the external standard population, was also reported. A sensitivity analysis was carried out to assess the impact of possible dementia on outcome misclassification. This was carried out by including head injury events that were not recalled by the participant, but that were documented during the informant interview.

Poisson regression models, estimating prevalence ratios (PR) were used to estimate the association of co-variables (age, gender, education level, number of assets and alcohol risk in mid-life) on the prevalence of head-injury. In order to estimate a pooled effect across sites, a meta-analysis was carried out, together with an estimation of heterogeneity using the Higgins I^1^. Fixed effect models were used, unless the heterogeneity from the I^2^ index was higher than 50%. In these circumstances a random-effect model was preferred.

Disability scores were measured using the WHODAS-12 scale, and modelled using zero-inflated negative binomial regressions (adjusted for age, gender and number of physical co-morbidities) to deal with over dispersion and excess zeros in the distribution of disability scores, as used in previous publications from the 10/66 group[25, 26].

## Results

### Socio-demographic characteristics

A total of 17,009 interviews were carried out and completed in 8 countries (Cuba, Dominican Republic, Venezuela, Mexico, Peru, India, China, and Puerto Rico). The overall response rate was very good with proportions higher than 80% in all but two centres: urban China (74%) and urban India (72%). Missing data on the variables of interest were present in less than 1% of the sample (**Table 1)**, with results for 16,925 participants reported here.

**Table 1:** Demographic and socioeconomic status information from the 10/66 study centres. MV = Missing values.

Study centre	n	Response rate (%)	Female,	Age					Education						Number of assets				
			n (%)	65–69	70–74	75–80	84+	MV	None	Minimal	Primary	Secondary	Tertiary	MV	0	1 to 3	4 to 5	6 to 7	MV
**Cuba**	2930	94%	1902 (64.9)	758 (25.9)	787(26.9)	635 (21.7)	742 (25.4)	7	75 (2.6)	653(22.9)	977 (33.4)	727 (24.8)	497 (17.0)	1	9 (0.3)	68 (2.3)	956 (32.7)	1889 (64.6)	8
**Dom. Rep.**	2009	95%	1325 (66.0)	533 (26.5)	519 (25.8)	397 (19.8)	560 (27.9)	0	392 (19.5)	1022 (51.3)	370 (18.6)	135 (6.8)	73 (3.7)	17	7 (0.3)	298 (14.9)	781 (38.9)	919 (45.8)	4
**Peru urban**	1372	80%	883 (64.4)	372 (27.1)	350 (25.5)	296 (21.6)	353 (25.8)	1	37 (2.7)	90 (6.6)	459 (33.6)	476 (34.8)	305 (22.3)	5	2 (0.1)	4 (0.3)	60 (4.4)	1306 (95.2)	0
**Peru rural**	550	88%	294 (53.4)	179 (32.5)	140 (25.4)	100 (18.2)	131 (23.8)	0	84 (15.3)	141 (26.0)	266 (49.0)	36 (6.6)	16 (2.9)	7	4 (0.7)	84 (15.3)	292 (53.1)	170 (30.9)	0
**Venezuela**	1894	80%	1200 (63.4)	815 (43.1)	455 (24.1)	334 (17.7)	287 (15.2)	3	147 (7.8)	442 (23.4)	946 (50.1)	262 (13.9)	90 (4.8)	7	36 (1.9)	0 (0.0)	9 (0.5)	1849 (97.6)	0
**Mexico urban**	1003	84%	666 (66.4)	245 (24.4)	329 (32.8)	205 (20.5)	223 (22.3)	1	227 (22.6)	354 (35.4)	229 (22.9)	99 (9.9)	92 (9.2)	2	2 (0.2)	36 (3.6)	125 (12.5)	840 (83.7)	0
**Mexico rural**	1000	86%	602 (60.2)	299 (29.9)	252 (25.2)	221 (22.1)	228 (22.8)	0	327 (32.7)	510 (51.0)	122 (12.2)	25 (2.5)	16 (1.6)	0	9 (0.9)	385 (3.5)	337 (33.7)	269 (26.9)	0
**China urban**	1160	74%	661 (57.0)	316 (27.2)	362 (31.2)	254 (21.9)	228 (19.7)	0	232 (20.0)	153 (13.2)	303 (26.12)	335 (28.9)	137 (11.8)	0	0 (0.0)	5 (0.4)	599 (51.7)	555 (47.9)	1
**China rural**	1002	96%	556 (55.5)	383 (38.2)	296 (29.5)	202 (20.2)	121 (12.1)	0	579 (57.8)	114 (11.4)	259 (25.9)	45 (4.5)	5 (0.5)	0	0 (0.0)	108 (10.8)	281 (28.0)	613 (61.2)	0
**India urban**	1004	72%	571 (57.7)	414 (41.4)	318 (31.8)	144 (14.4)	124 (12.4)	4	428 (42.6)	234 (23.3)	212 (21.1)	87 (8.7)	42 (4.2)	1	13 (1.3)	351 (35.1)	388 (38.8)	248 (24.8)	4
**India rural**	999	92%	545 (54.5)	331 (33.1)	350 (35.0)	177 (17.7)	141 (14.1)	0	660 (66.1)	195 (19.5)	116 (11.6)	26 (2.6)	2 (0.2)	0	66 (6.6)	618 (61.9)	272 (27.2)	43 (4.3)	0
**Puerto Rico**	2002	93%	1347 (67.3)	411 (20.5)	455 (22.7)	483 (24.1)	653 (32.6)	0	72 (3.6)	389 (19.4)	415 (20.7)	713 (35.6)	410 (20.5)	0	3 (0.1)	1 (0.1)	38 (1.9)	1960 (97.9)	0
**Total**	16925		10552 (62.3)	5056 (29.9)	4613 (27.3)	3448 (20.4)	3791 (22.4)	16	3260 (19.3)	4297 (25.4)	4674 (27.6)	2966 (17.5)	1685 (9.96)	40	147 (0.01)	1958 (0.1)	4138 (24.4)	10661 (63.0)	17

Across all 10/66 study sites, there were more women than men with an overall ratio of 60.7% females and 39.3% males, as seen in **Table 1**. Most participants were either married or widowed, and this trend was consistent across all centres. Between 50–70% of participants in all sites were aged between 65 and 74 years. There was a higher proportion of people aged 80 years or more in Latin America and the Caribbean (especially in Cuba, the Dominican Republic, Peru, and Puerto Rico) compared to Asia, indicating a more advanced demographic shift. Urban Peru and Puerto Rico had the highest proportion of tertiary educated participants (>20%), while rural India and rural China had the lowest proportion (<1%). In rural China and rural India, respectively 57.8% and 66.1% of the population did not have any education. Generally, these numbers were lower in Latin America, with 2.6%-32.7% having no education.

### Prevalence of head injury across the 10/66 sites

The prevalence of head injury across 10/66 sites is shown in **Table 2**. Rural and Urban Mexico (16.4% and 15.7% respectively), rural Peru (15.3%), and Venezuela (14.3%) had the highest crude prevalence of TBI across the 10/66 sites, while urban China (0.9%) and rural China (0.4%) had the lowest. Except for urban China and urban India the crude prevalence in males was significantly higher than in females with the highest difference in rural Mexico (males 26.1%, females 10.0%,). Direct age, sex and education standardisation of the prevalence did not greatly affecte the estimates, with the two Mexican centres and Venezuela still having the highest prevalence of head injury (between 14.4% and 14.6%).

**Table 2:** Crude and age, sex, and education-standardized prevalence of self-reported head injury in the 10/66 study centres.

Study Centre	Number of cases	Crude Prevalence (95%CI)	Crude Prevalence In Males (95%CI)	Crude Prevalence In Females (95%CI)	Standardised Prevalence (95%CI)	Mean age at most recent head injury (SD)
**Cuba**	186	6.3 (5.5–7.3)	9.1 (7.5–11.1)	4.8 (3.9–5.9)	5.9 (4.8–7.0)	41.7 (26.3)
**Dom. Rep.**	225	11.2 (9.9–12.6)	14.5 (12.1–17.3)	9.5 (8.0–11.2)	11.0 (9.0–12.8)	53.4 (25.2)
**Peru urban**	186	13.6 (11.8–15.5)	18.2 (15.1–21.8)	11.0 (0.9–13.3)	12.2 (9.4–15.0)	37.6 (25.2)
**Peru rural**	84	15.3 (12.4–18.6)	16.0 (12.0–21.0)	14.6 (11.1–14.8)	13.7 (10.9–16.5)	37.3 (24.7)
**Venezuela**	271	14.3 (12.8–16.0)	16.9 (14.3–19.8)	12.8 (11.1–14.8)	14.6 (12.3–16.8)	34.9 (23.8)
**Mexico urban**	157	15.7 (13.5–18.0)	26.1 (21.7–31.0)	10.4 (8.3–12.9)	14.4 (12.2–16.7)	43.1 (43.1)
**Mexico rural**	164	16.4 (14.2–18.9)	26.1 (22.1–30.6)	10.0 (7.8–12.6)	14.6 (11.5–17.8)	48.2 (23.4)
**China urban**	11	0.9 (0.5–1.7)	0.8 (0.3–2.1)	1.1 (0.5–2.2)	1.1 (0.4–1.9)	61.2 (14.3)
**China rural**	4	0.4 (0.01–0.8)	0.7 (0.2–2.1)	0.2 (0.2–1.3)	0.3 (0.0–0.6)	69.2 (8.9)
**India urban**	98	9.8 (8.0–11.8)	9.5 (7.1–12.8)	10.0 (7.8–12.7)	9.3 (7.0–11.5)	44.4 (24.7)
**India rural**	52	5.2 (4.0–6.8)	6.4 (4.5–9.0)	4.2 (2.8–6.3)	2.9 (1.7–4.1)	63.9 (14.3)
**Puerto Rico**	98	4.9 (4.0–5.9)	6.0 (4.4–8.0)	4.4 (3.4–5.6)	4.8 (3.5–6.1)	52.4 (23.4)
**Overall sample**	1,563	9.1 (8.6–9.5)	11.7 (11.0–12.5)	7.5 (7.0–8.0)		44.2 (25.3)

**Fig 1:**
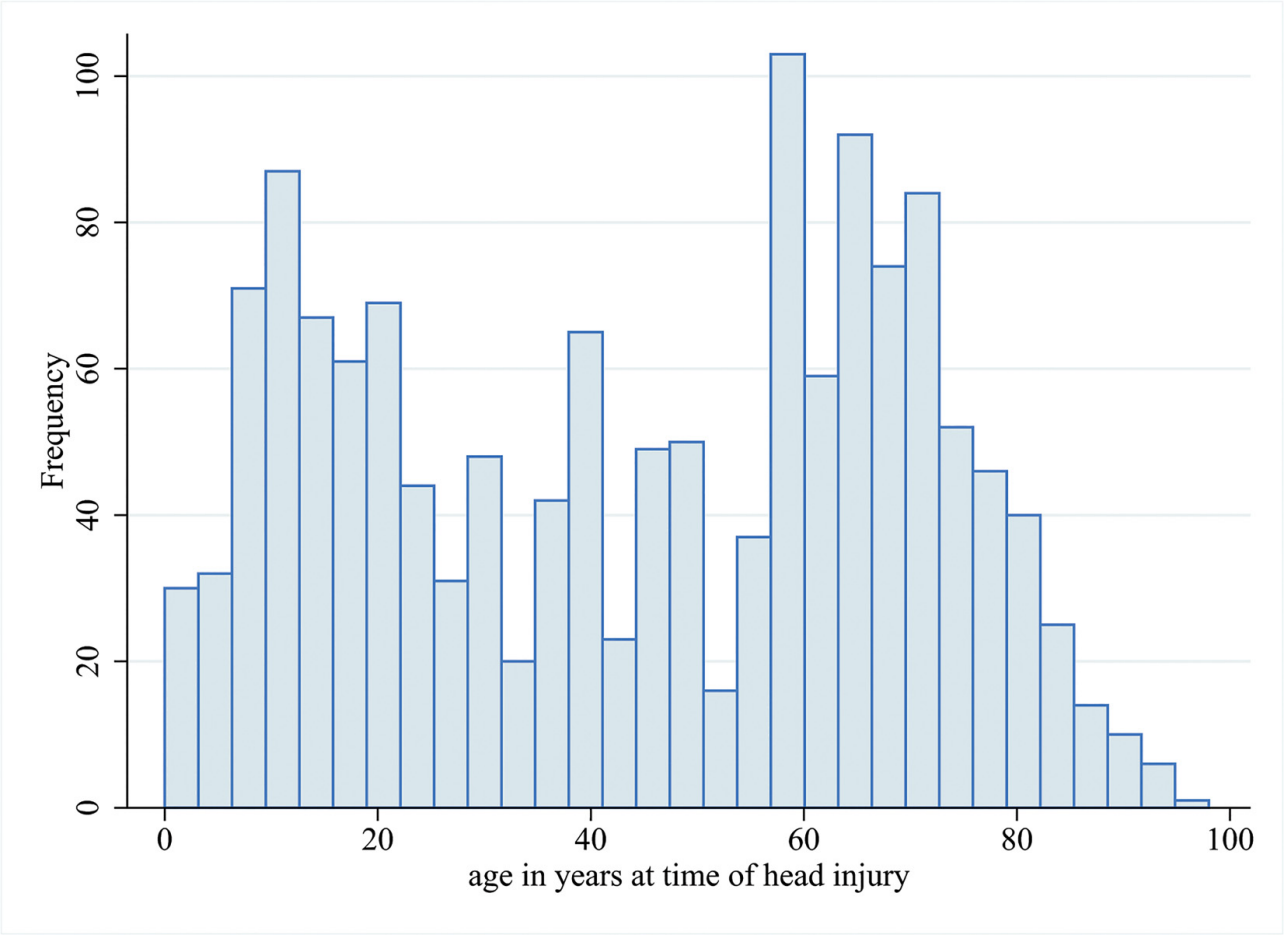
Distribution of age at time of head injury.

We found a bi-modal distribution of age of head injury with a peak during adolescence and a second one around age 60 (**Fig 1.**).

### Effect of using informant interview on estimates

**S1 Fig** shows the results of the sensitivity analysis and the proportional increase in estimates when data from an informant was used to estimate the prevalence of head injury (informants were used when participants screened positive for possible dementia). In most cases, this increase was null or below 1%. Only in Venezuela, Mexico and the Dominican Republic, was there an increase of between 1% and 2% in the estimates.

### Independent association of co-variables and head-injury prevalence

To investigate the association of head-injury with potential risk factors, a Poisson regression model was used to provide mutually adjusted prevalence ratios, as illustrated in **Table 3**. The results indicate that the prevalence of TBI was strongly related to gender, and that being male consistently increased the chance of having TBI across all centres after adjustment for age, education, number of assets and alcohol problems in mid-life (pooled PRs across sites: 1.56–95% CI: 1.29–1.82, I^2^ = 52.5%). The magnitude of association was higher in rural sites compared to urban sites, with the exception of rural Peru.

**Table 3:** Association of risk factors for head injury by country with pooled estimates.

Study Centre	Age	95% CI	Gender	95% CI	Education	95% CI	Assets	95% CI	Alcohol problems in midlife	95% CI
**Cuba**	0.90	(0.80–1.01)	1.80	(1.34–2.43)	1.08	(0.94–1.25)	1.01	(0.85–1.19)	1.17	(0.74–1.83)
**Dominican Rep.**	1.02	(0.93–1.12)	1.29	(0.97–1.73)	0.96	(0.83–1.11)	0.88	(0.81–0.95)	1.33	(0.99–1.78)
**Peru urban**	0.96	(0.87–1.06)	1.61	(1.23–2.13)	1.06	(0.91–1.22)	1.08	(0.85–1.36)	0.90	(0.32–2.54)
**Peru rural**	0.95	(0.82–1.11)	1.15	(0.75–1.76)	0.77	(0.62–0.96)	0.92	(0.80–1.06)	2.35	(1.10–5.04)
**Venezuela**	0.87	(0.77–0.99)	1.42	(1.05–1.92)	0.85	(0.73–0.98)	0.91	(0.82–1.01)	1.08	(0.66–1.79)
**Mexico urban**	1.05	(0.94–1.16)	2.36	(1.74–3.20)	0.85	(0.75–0.97)	1.01	(0.88–1.17)	1.32	(0.90–1.93)
**Mexico rural**	0.94	(0.84–1.04)	2.80	(2.07–3.81)	0.91	(0.76–1.08)	0.92	(0.86–0.99)	0.87	(0.59–1.28)
**China urban**	1.36	(0.80–2.31)	0.67	(0.14–3.20)	0.83	(0.48–1.42)	1.35	(0.63–2.93)	5.79	(0.66–50.48)
**China rural**	1.80	(1.07–3.05)	4.27	(0.36–50.1)	0.79	(0.34–1.86)	1.34	(0.58–3.10)	4.65	(0.34–63.4)
**India urban**	0.99	(0.85–1.17)	1.09	(0.72–1.63)	0.81	(0.66–0.99)	1.01	(0.93–1.28)	2.86	(0.45–18.15)
**India rural**	0.83	(0.62–1.10)	1.88	(1.05–3.37)	0.86	(0.59–1.25)	0.87	(0.72–1.05)	Too few cases	
**Puerto Rico**	0.90	(0.77–1.05)	1.46	(0.95–2.23)	1.03	(0.86–1.24)	1.53	(0.90–2.62)	0.48	(0.17–1.37)
**Pooled estimates** *	**0.95**	**(0.92–0.99)**	**1.56**	**(1.29–1.82)**	**0.91**	**(0.86–0.96)**	**0.92**	**(0.88–0.96)**	**1.07**	**(0.88–1.25)**
**Higgins I** ^**2**^	20.5%		52.5%		33.6%		0.0%		0.0%	

* **Pooled estimates from a fixed effect model, apart from where the heterogeneity was higher than 50%.**

There was a mild inverse association between age at survey interview and head injury, with a pooled effect of 0.95% (95% CI: 0.92–0.99). Socio-economic factors, such as education and number of assets were both associated with prevalent head-injury. Having spent more years in education was a protective factor for head-injury in most sites, with a pooled prevalence ratio of 0.91 (95% CI: 0.86–0.96). Similarly, owning a higher number of assets was associated with a lower prevalence of head injury. This was however not statistically significant across all sites, but the pooled estimate was still statistically significant (0.92, 95% CI: 0.88–0.96). Finally alcohol problems in midlife were not found to be related to head injury, with the exception of rural Peru, where the prevalence ratios for people with alcohol problems were 2.35 (95% CI: 1.10–5.04) higher than people without alcohol problems in midlife.

### The impact of head injury

Mean WHODAS-12 disability scores were stratified by head injury status. Participants with a self-reported brain injury had consistently higher mean disability scores, compared with people without a reported head injury, with the exception of the Cuban site (**Table 4)**. The scores were modelled using a zero-inflated negative binomial regression to take into account the effect of potential confounding variables (age at survey interview, gender and co-morbid physical illnesses) on this association. In Venezuela, China, urban India and Puerto Rico, the association between disability and head injury was still significant after adjustment for potential confounders, with a pooled estimate across all 10/66 sites of 1.11 (95% CI: 1.05–1.18) (**Table 4**).

**Table 4:** WHODAS Disability scores by head-injury status and country.

Centre		Mean (SD) WHODAS Disability scores	Adjusted* RR (95% CI)
**Cuba**	No Head Injury	13.40 (20.10)	
	Head Injury	12.75 (18.66)	1.00 (0.81–1.25)
**Dominican Rep.**	No Head Injury	15.86 (19.81)	-
	Head Injury	21.45 (23.25)	1.09 (0.96–1.25)
**Peru urban**	No Head Injury	12.96 (20.65)	-
	Head Injury	13.56 (19.44)	0.91 (0.75–1.10)
**Peru rural**	No Head Injury	9.67 (14.27)	-
	Head Injury	14.61 (15.52)	1.17 (0.91–1.51)
**Venezuela**	No Head Injury	10.21 (16.42)	-
	Head Injury	14.08 (15.95)	1.23 (1.07–1.41)
**Mexico urban**	No Head Injury	9.46 (17.10)	-
	Head Injury	12.72 (17.98)	1.08 (0.85–1.37)
**Mexico rural**	No Head Injury	10.72 (18.71)	-
	Head Injury	12.91 (20.95)	1.00 (0.79–1.26)
**China urban**	No Head Injury	7.80 (19.55)	-
	Head Injury	38.38 (45.92)	1.79 (1.18–2.70)
**China rural**	No Head Injury	7.85 (14.30)	-
	Head Injury	43.75 (32.02)	1.85 (1.00–3.42)
**India urban**	No Head Injury	9.96 (14.85)	-
	Head Injury	15.82 (19.37)	1.34 (1.06–1.71)
**India rural**	No Head Injury	28.19 (18.03)	-
	Head Injury	29.54 (22.39)	1.16 (0.97–1.38)
**Puerto Rico**	No Head Injury	16.17 (22.44)	-
	Head Injury	25.23 (28.31)	1.25 (1.03–1.51)
**Pooled estimate**			**1.11 (1.05–1.18) I** ^**2**^ **= 35.2%**

*RR from a zero-inflated negative binomial regression, adjusted for age, gender and number of co-morbid physical illnesses.

This analysis was also stratified according to age of injury, using no head injury as the control category. An association between head injury after age 65 and disability was found in the adjusted model (pooled estimate RR: 1.17, 95% CI: 1.05–1.28), but no significant associations were found between disability and head injury before age 65 (pooled RR = 1.01, 95% CI, 0.93–1.21) (**S1 Table**).

## Discussion

This study reported that the prevalence of head injury with loss of consciousness is common in the 10/66 centres (mean = 9.1, range from 3.2% to 13.6%), with the exception of China. Being male, having a lower number of assets, and lower education were associated with a higher prevalence of head injury. Disability scores in people with head injury were higher than in people who did not report any head injury, even after adjustment for age, gender and number of co-morbid physical illnesses.

### Strengths and Limitations

A key strength of the 10/66 project is that it provides a large dataset, which was designed specifically to investigate ageing-related public health issues in LMICs. Apart from covering 17,009 participants over 8 LMICs, data collected at the study sites encompassed a wide variety of variables, with very low rates of missing data. Since interpretation and model development of this dataset relies crucially on the accuracy of the information gathered, the methods were adapted, validated, and translated into different languages, enabling comparability between different countries.

A major advantage of using 10/66 study data, especially when comparing trends in different countries, is that all data were systematically collected using identical standardised protocols. This provides a firm basis for analysing the prevalence of head injury and its potential association with risk factors.

The main limitations of the study are that firstly head injury ascertainment was accomplished through participants self-report, and secondly the use of lifetime prevalence of head injury as an outcome, which did not record and take into account multiple episodes of head injury. These approaches may be misleading as they can lead to recall bias of outcome as one cannot be sure if/when TBI actually happened, especially if it occurred more than a few months or a year before the ascertainment. If people do not remember correctly, there will be non-differential misclassification of the outcome with respect to the exposures. It is possible that people with dementia under-reported head injury, which would result in an underestimation of its prevalence. This seems to be confirmed by our sensitivity analysis that showed some discrepancy between informant and participant reports in people with possible dementia. Using informant reports resulted in a slight increase in prevalence estimates. Although the analyses were adjusted for possible confounders such as age, sex, education, number of assets, and alcohol problems in mid-life, some residual confounding may be remaining.

Finally, it is difficult to know the exact generalisability of these findings outside these catchment areas, and the direction of association with risk factors and disability due to the cross-sectional nature of the study design.

### Contextualisation

A recent meta-analysis estimated the lifetime prevalence of TBI with loss of consciousness in general populations to be around 12%[5], however these data come from high-income countries. In comparison with the current results, this implies that the prevalence of head injury in low and middle income countries may be similar to that in high income countries.

Most TBI prevalence data in the literature have been estimated from hospitalised incidence statistics. This is not necessarily comparable to the 10/66 study which derives the prevalence directly from individuals interviewed from population settings, regardless of whether they sought medical attention or not. This is particularly relevant for LMICs, which do not have the same medical coverage as HICs.[27]

The analysis in the current study used participant questionnaires (and informant data for people with possible dementia), whereas other studies have predominantly used medical records, or a combination of all sources[28–37]. Although informants may be reliable sources compared to subjects with dementia, who may be at risk of not recalling TBI, informants themselves may not be aware of a head injury history, and this is why we only included their reports in our sensitivity analysis. The use of medical records, one of the best ways of measuring head injury accurately, also has some limitation as it increases the risk of incorporating measurement bias into the analysis, as records may not be complete, or methods of data collection may have differed across place and time, especially if done in the distant past. Moreover, not all the individuals who suffer a head injury may visit hospitals or emergency departments. This is particularly relevant for low and middle-income countries where medical records are not often routinely collected and where their quality can be poor.

Another important factor that may affect the prevalence of TBI is the setting from which the sample was selected. Several studies have been based in clinical settings, such as hospital departments, and neurological clinics.[28–30, 33, 35, 38, 39], compared to our population-based sample. In clinical settings it is not possible to know what referral processes or access differences may exist and hence these differences will not necessarily reflect the situation in the general population.

After adjusting for other risk factors, both gender and education showed a statistically significant positive association with TBI in the 10/66 cohort. The association with gender is in line with the current literature indicating that men are at greater risk of TBI, with men reported to have ORs of 2.2 for traumatic brain injury in a recent meta-analysis of published studies[5]. The finding that men are at higher risk of head injury may be due to differential societal roles of men and women in different settings. In many low and middle income countries, men often have manual jobs, have a higher tendency to exhibit more risk taking behaviour and are consequently at higher risk of injury. Conversely, women may look after the home, where their risk of injury may be lower. Given that traditional societal roles are slowly changing across the world, with women increasingly working outside the home settings, it is possible that risk of TBI in women may increase in the future too. In our analysis we could not see an association between head injury and abnormal alcohol use in later life, but this is likely due to be the result of low statistical power. While alcohol intoxication has been reported to be a risk factor for head injury in previous analysis[7], reported later life alcohol use in our study may be may be an outcome of having experienced a TBI and a mechanism of coping with the effect of the injury.

In this study, we did not see any significant difference in head injury prevalence between urban and rural sites, with the exception of India where the urban site had almost a two-fold higher prevalence of TBI, compared to rural one. It is possible that this is due to a higher rate of road traffic accidents in urban settings. Previous research carried out in India has reported that people in urban settings had 1.5 higher OR of road traffic injury deaths, compared to those living in rural areas[40]. In contrast to HICs, where the drivers and passengers of vehicles sustain TBI, in LMICs pedestrians, cyclists and motorcyclists are more vulnerable to sustaining TBI. This is because there has been an increase in the number of motor vehicles, which has not been addressed by suitable road safety education and regulations[9].

Both urban and rural centres in China reported extraordinarily low prevalence of TBI compared to all other sites. While the rates of road traffic crashes in this country are among the highest in the world[41], this may not have been true during the early periods of the older people included in our study. These results could be due to cultural factors affecting reporting behaviour, or some ascertainment bias, where interviewers did not realise or did not stress that we were asking about lifetime exposure.

Finally we found a significant association between disability and head injury, in line with the global burden of disease project, which highlights traumatic brain injury as a leading cause of disability across all the regions of the globe. When we stratified this analysis by age of injury we only found a significant association in those with a head injury after age 65. It is however difficult to interpret these results as the statistical power in each site was low, and the age of head injury was also highly susceptible to recall bias.

### Conclusions

TBI is a major and growing public health problem, afflicting an estimated 12% of the global population[5]. There is very little known about the prevalence and impact this may have on the public health situation, particularly in LMICs. This study has highlighted that TBI is prevalent at levels as high as HICs. Road traffic accidents are the leading cause of TBI injury in low and middle income countries, compared to falls in the home, which are the main cause in high-income countries. Increasing use of motor vehicles in these settings will exacerbate this trend[6]. This highlights the need for more studies to be carried out in low and middle income-settings.

Taking examples of prevention strategies from HICs and applying them in LMICs may also be useful. For example, improved safety regulations in HICs have resulted in a decline in TBI from road traffic accidents[42]. Road traffic related injury could be reduced by improving, promoting, and legislating different aspects of safety, such as the use of seat-belts, reducing the number of people under the influence of alcohol or drugs, and promoting the use of helmets during cycling and contact sports. Implementation of helmet use in Taiwan has proven beneficial, resulting in a 33% reduction in motorcycle accident related TBI[43]. Given that falls are a major cause of TBI in older and younger people, promotion of better engineering and design of private and public spaces (e.g. removal of tripping hazards, use of railings for stability, etc) could also help in reducing TBI.

At present, similar to other clinical conditions, there is no consensus on a classification system. Usually TBI is diagnosed when the symptoms are closely temporally related to the incident that caused the injury, although clinical manifestations can occur at a delayed time. Other evidence of brain pathology from imaging, or diagnostic confirmation of damage to the brain, should ideally be used for confirmation[44], but these may not be feasible in poorer settings or those that have lower resources.

Considering the predicted future impact of TBI on public health and resources, there is a need for further research and understanding of the problem with a view to informing public health strategies that could reduce the rate of TBI.

#### Ethics

Written informed consent, or witnessed oral consent in case of illiteracy, or next of kin written agreement in case of incapacity, was obtained from all participants. The study protocol and the consent procedures were approved by the King's College London research ethics committee and in all local countries: 1- Medical Ethics Committee of Peking University the Sixth Hospital (Institute of Mental Health, China); 2- the Memory Institute and Related Disorders (IMEDER) Ethics Committee (Peru); 3- Finlay Albarran Medical Faculty of Havana Medical University Ethical Committee (Cuba); 4- Hospital Universitario de Caracas Ethics Committee (Venezuela); 5- Ethics Committee of Nnamdi Azikiwe University Teaching Hospital (Nigeria); 6- Consejo Nacional de Bioética y Salud (CONABIOS, Dominican Republic); 7- Christian Medical College (Vellore) Research Ethics Committee (India); 8- Instituto Nacional de Neurología y Neurocirugía Ethics Committee (Mexico).

## Supporting Information

S1 Fig The effect of including informant information in people with cognitive impairment on the estimates of head injury. (PDF)

S1 Table The association of disability and head injury before and after age 65. *RR from a zero-inflated negative binomial regression, adjusted for age, gender and number of co-morbid physical illnesses. ~ reference is no head injury.
(DOCX)
