# Supplementary figures and images for: Lifetime Prevalence and Factors Associated with Head Injury among Older People in Low and Middle Income Countries: A 10/66 Study
Source: PLoS One. 2015 Jul 6;10(7):e0132229. doi: 10.1371/journal.pone.0132229 (PMC4493012; doi:10.1371/journal.pone.0132229)

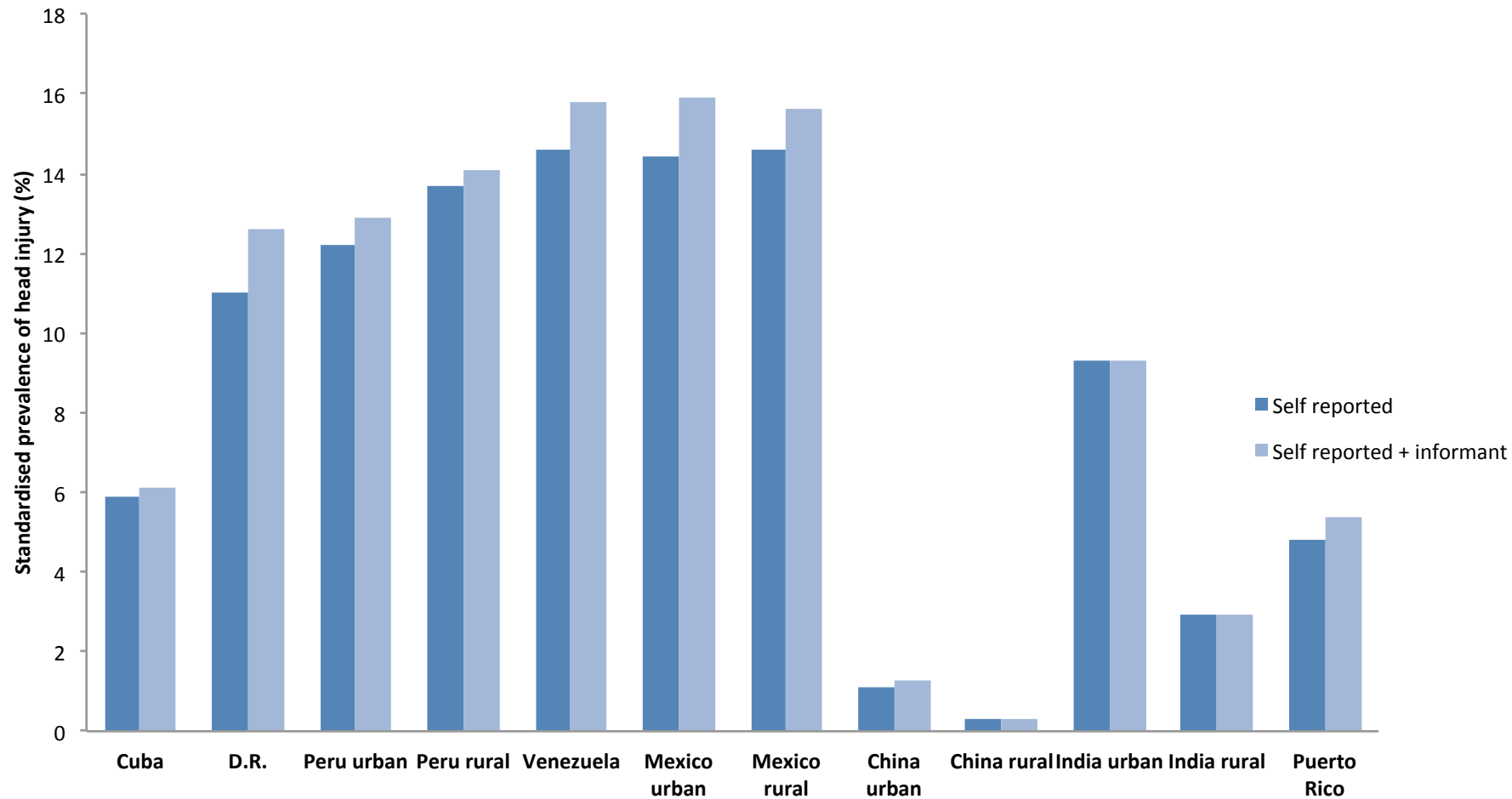

Supplement: S1 Fig — (PDF) [file pone.0132229.s001.pdf]
