# Supplementary material for: Lifetime Prevalence and Factors Associated with Head Injury among Older People in Low and Middle Income Countries: A 10/66 Study
Source: PLoS One. 2015 Jul 6;10(7):e0132229. doi: 10.1371/journal.pone.0132229 (PMC4493012; doi:10.1371/journal.pone.0132229)
Supplement: S1 Table — *RR from a zero-inflated negative binomial regression, adjusted for age, gender and number of co-morbid physical illnesses. ~ reference is no head injury. (DOCX) [file pone.0132229.s002.docx]

| **Centre** | **Head injury~** | **Adjusted* RR**  **(95% CI)** |
| --- | --- | --- |
| **Cuba** | Before age 65 | 0.83 (0.65-1.06) |
|  | After age 65 | 1.38 (0.97-1.97) |
|  |  |  |
| **Dominican Rep.** | Before age 65 | 1.02 (0.86 – 1.22) |
|  | After age 65 | 1.20 (0.97 – 1.49) |
|  |  |  |
| **Peru urban** | Before age 65 | 0.93 (0.75 – 1.17) |
|  | After age 65 | 0.90 (0.64 – 1.28) |
|  |  |  |
| **Peru rural** | Before age 65 | 1.14 (0.88 – 1.50) |
|  | After age 65 | 1.54 (0.84 – 2.82) |
|  |  |  |
| **Venezuela** | Before age 65 | 1.12 (0.94 – 1.32) |
|  | After age 65 | 1.46 (1.13 – 1.90) |
|  |  |  |
| **Mexico urban** | Before age 65 | 1.11 (0.84 – 1.48) |
|  | After age 65 | 0.91 (0.61 – 1.36) |
|  |  |  |
| **Mexico rural** | Before age 65 | 0.85 (0.63 – 1.13) |
|  | After age 65 | 1.12 (0.79 – 1.59) |
|  |  |  |
| **China urban** | Before age 65 | 1.49 (0.81 – 2.75) |
|  | After age 65 | 1.62 (0.85 – 3.09) |
|  |  |  |
| **China rural** | Before age 65 | 1.36 (1.18 – 1.55) |
|  | After age 65 | 2.02 (0.97 – 4.20) |
|  |  |  |
| **India urban** | Before age 65 | 1.38 (1.04 – 1.81) |
|  | After age 65 | 1.26 (0.82 – 1.94) |
|  |  |  |
| **India rural** | Before age 65 | 1.15 (0.91 – 1.46) |
|  | After age 65 | 1.16 (0.92 – 1.48) |
|  |  |  |
| **Puerto Rico** | Before age 65 | 1.31 (1.03 – 1.66) |
|  | After age 65 | 1.17 (0.87 – 1.60) |
| **Pooled estimate** | **Before age 65** | **1.01 (0.93 – 1.21)** |
|  | **After age 65** | **1.17 (1.05 – 1.28)** |
|  | |  |

S2 Table - The association of disability and head injury before and after age 65. *RR from a zero-inflated negative binomial regression, adjusted for age, gender and number of co-morbid physical illnesses. ~ reference is no head injury.
